# Renal endometriosis: A benign disease with malignant presentation

**DOI:** 10.1016/j.eucr.2022.102110

**Published:** 2022-05-07

**Authors:** Muhammad Mubashir Nawaz, Yasir Masood, Afshan Saeed Usmani, Muhammad Irfan Basheer, Umer Nisar Sheikh, Khurram Mir

**Affiliations:** Shuakat Khanum Memorial Cancer Hospital and Research Center Lahore, Pakistan

**Keywords:** Renal endometriosis, Urinary tract endometriosis, Nephrectomy, Malignant presentation

## Abstract

Endometriosis is a common gynecological disorder in which endometrial tissue is located outside the uterine cavity. Urinary tract involvement by endometriosis is uncommon. Renal endometriosis is a rare disorder and is not evident on imaging. It may be misdiagnosed as malignant disease and patients may undergo invasive procedures for it. We report a case of a young lady who presented with symptoms typical for renal mass, and was diagnosed as a tumor on imaging. Patient underwent radical surgery and histology revealed renal endometriosis.

## 1 Introduction

Endometriosis is the abnormal implantation of normal endometrial mucosa other than the uterine cavity. It is broadly categorized into endopelvic and extrapelvic disease.^1^ Uterosacral ligaments posterior of uterus, fallopian tube, ovaries and minor pelvis fall under endopelvic category whereas, Extra pelvic sites include scars in the perineum, abdominal wall, the thorax, nasal mucosa, urinary and the gastrointestinal tract. The incidence rate varies from 8% to 10% in women of reproductive age group. Chronic pelvic pain is the most common manifestation of endometriosis.^2^

Incidence of urinary tract endometriosis is 1–2%. Bladder and ureter endometriosis comprise of 85% and 15% respectively while renal and urethral endometriosis is approximately less than 1% of all cases.^3^ Flank pain, hematuria and renal mass are the most common presentation of renal endometriosis and it is misdiagnosed as renal cell carcinoma on imaging. Evidence shows that urinary tract endometriosis is uncommon but cases are reported, where usually it is misdiagnosed as renal cancer.^4^ The aim of this report is to share our experience of a case of renal endometriosis.

## 2 Case report

A 30-year-old female presented in an outpatient clinic with left sided flank pain with no associated lower urinary tract symptoms. She had a normal menstruation cycle. Her past medical history was significant for asthma while surgical history was unremarkable. No abnormality was noted on physical examination.

**Fig. 1:**
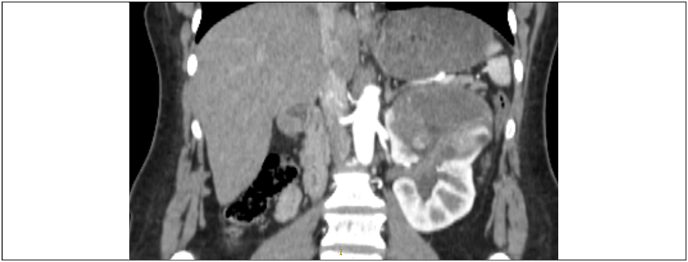
Coronal view of left renal tumor.

**Fig. 2:**
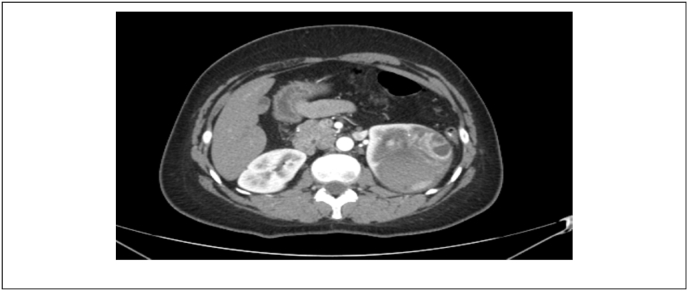
Axial view of left renal tumor.

**Fig. 3:**
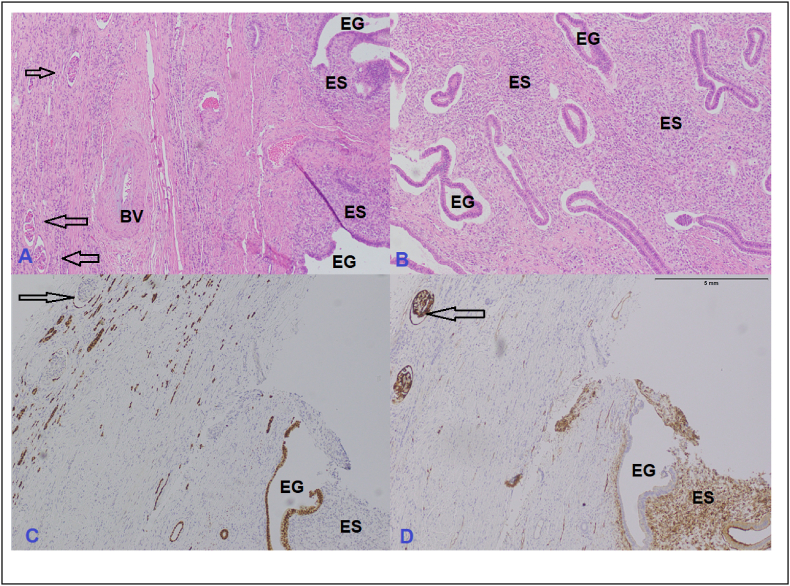
A: Hematoxylin and Eosin (H&E) stained image depicting renal tissue (arrows indicating renal glomeruli) and a thick-walled Blood vessel (BV) on the left side and Endometrial glands (EG) surrounded by bluish endometrial stroma (ES) on the right side of the image, B: Another H&E image from the same patient showing numerous endometrial glands surrounded by endometrial stroma, C: Estrogen Receptor antibody highlighting nuclear positive staining of endometrial glands, D: CD10 antibody showing cytoplasmic staining in cells of endometrial stroma surrounding the glands.

Laboratory investigations showed Hb of 14g/l, serum creatinine of 0.4mg/dl and an estimated glomerular filtration rate >100 (mL/min/1.73 m^2^). USG abdomen revealed left renal mass at upper pole. Contrast CT scan showed a 7.7 cm unifocal multilocular cystic mass over upper pole of the left kidney with extension into renal pelvis. Presence of nodular enhancing component along the lateral wall of lesion, foci of calcification, and extension into the renal pelvis were concerning. These features made the lesion Bosniak type 4 cyst which has significant potential for malignancy (Fig. 1, Fig. 2). Metastatic work-up was negative. We discussed the case in a multidisciplinary tumor board meeting, and it was decided that since renal lesion is quite concerning for malignancy and our differential may include urothelial carcinoma of renal pelvis given the features of the tumor. Open nephroureterectomy with cuff of bladder was performed under general anesthesia. Patient had an uneventful recovery and was discharged on POD-4. Her histopathology report revealed a well-circumscribed lesion consisting of endometrial type stroma and glands consistent with endometriosis (Fig. 3).

## 3 Discussion

Urinary tract involvement by endometriosis is rare and reported cases in literature are few in number. Renal endometriosis is an infrequent disease and its pathological mechanism is not well understood.^3^

Hematuria, flank pain and renal colic lead to a variety of urological differentials, and renal endometriosis is usually not considered in differentials because of its rarity. Moreover, imaging features of renal endometriosis do not demonstrate an accurate diagnosis. It cannot be distinguished from cystic renal malignancy on ultrasound, CT scan or MRI. These patients undergo surgery because the clinical scenario favors malignancy.^4^

Renal endometriosis should be considered when the patient is in the reproductive age group and symptoms are cyclical in nature correlating with menses. Treatment options vary from active surveillance to thermal ablation, partial or radical nephrectomy depending upon the complexity of the tumour.^5^ In our case, the patient had symptomatic presentation with the tumor extending into renal pelvis mimicking a urothelial malignancy. Patient underwent a nephroureterectomy. Patient recovered without complication post operatively and was asymptomatic on her follow-up visit in the clinic.

## 4 Conclusion

We present a case of a young female with renal endometriosis, presented with flank pain only, and diagnosed as renal pelvic cystic tumor. We recommend that endometriosis should be considered in females of childbearing age with unusual symptoms of renal tumor.
